# Endocrine Tumors: Diagnosis, Treatment, and Management—Closing Editorial

**DOI:** 10.3390/jcm14248890

**Published:** 2025-12-16

**Authors:** Jules Zhang-Yin, Emmanouil Panagiotidis, Francesco Dondi

**Affiliations:** 1 Department of Nuclear Medicine, Clinique Sud Luxembourg, 6700 Arlon, Belgium; 2 Department of Nuclear Medicine, Centre National Positron Emission Tomography, L-1210 Luxembourg, Luxembourg; 3 Nuclear Medicine Laboratory, University Hospital of Larissa, University of Thessaly, 41110 Larissa, Greece; m_panagiotidis@yahoo.com; 4 Nuclear Medicine, Azienda Socio Sanitaria Territoriale Spedali Civili di Brescia, Università degli Studi di Brescia, 25121 Brescia, Italy; f.dondi@outlook.com

Endocrine tumors encompass a diverse group of neoplasms arising from hormone-producing glands, including the thyroid, parathyroid, pituitary, adrenal glands, and neuroendocrine cells dispersed in organs like the pancreas and gastrointestinal tract [1,2]. They range from common entities with generally good prognosis (e.g., differentiated thyroid carcinoma) to exceptionally rare and aggressive malignancies (e.g., anaplastic thyroid carcinoma or parathyroid carcinoma) [1,2]. Managing these tumors presents unique challenges: not only must the tumor itself be controlled, but often the hormonal disturbances they cause (such as thyroid hormone excess, cortisol excess in Cushing’s disease, or growth hormone excess in acromegaly) require simultaneous treatment. In launching this Special Issue, we aimed to provide a comprehensive platform for researchers and clinicians to share the latest advancements in the diagnosis, treatment, and long-term management of endocrine tumors, highlighting novel insights and placing current research in a broader context. Now, as the Special Issue concludes, we reflect on the contributions and emerging themes that shed light on the state of the art in this field.

Accurate and early diagnosis is crucial for improving outcomes in endocrine tumors. Many of these tumors are small and asymptomatic in early stages or discovered incidentally, yet timely identification can greatly affect management. Traditional diagnostic tools like biochemical assays and cytology remain indispensable. For example, fine-needle aspiration biopsy of thyroid nodules has long been the gold standard to distinguish benign from malignant nodules, helping to avoid unnecessary thyroid surgeries while ensuring cancers are not missed. Recent studies in this Special Issue underscore the refinement of risk stratification in thyroid nodule evaluation. Krzentowska et al. identified that nodules falling into higher-risk cytological categories (e.g., Category V in diagnostic classification) carry a high likelihood of malignancy, warranting prompt surgical intervention, especially in younger patients [3]. In contrast, tumors in indeterminate categories (III and IV) showed lower malignancy rates, suggesting that close surveillance can be an appropriate strategy in select cases [3]. Such nuanced findings support a more individualized approach to diagnosing and managing thyroid nodules, balancing the risks of surgery against active surveillance in borderline cases. This approach contributes to optimizing the management of thyroid nodules, avoids unnecessary surgery, and aligns with the latest international guidelines [4].

Imaging modalities have dramatically advanced the detection and staging of endocrine tumors. High-resolution ultrasound and cross-sectional imaging (CT/MRI) are routine in evaluating thyroid, parathyroid, and adrenal lesions. Moreover, positron emission tomography/computed tomography (PET/CT) has emerged as a valuable adjunct in certain contexts. While [18F]FDG PET/CT is not typically used for initial evaluation of well-differentiated thyroid cancers due to their often indolent and low-metabolism nature, it plays an essential role in detecting recurrence and metastatic disease—for instance, identifying cancer foci in patients with elevated thyroglobulin but negative radioiodine scans. In this Special Issue, Panagiotidis and Zhang-Yin provided a comprehensive review of the current applications of PET/CT in differentiated thyroid carcinoma, highlighting how this metabolic imaging modality can guide management in challenging scenarios such as radioiodine-refractory disease. Similarly, functional imaging is invaluable in neuroendocrine tumors (NETs), where specialized scans (e.g., [68Ga]DOTATATE PET for somatostatin receptor imaging) help localize hormone-receptor-expressing tumors and metastases [5].

Beyond imaging and cytology, molecular diagnostics are increasingly informing the workup of endocrine tumors. Advances in genomics and molecular biology have significantly enhanced our understanding of these neoplasms. For thyroid cancers, well-characterized mutations in genes like BRAF, RAS, and RET are being used to refine diagnostic algorithms and even guide targeted therapies. In the realm of parathyroid tumors, genetic insights are especially illuminating, and parathyroid carcinoma, though exceedingly rare, has been linked to defects in the CDC73 tumor suppressor gene (encoding parafibromin). Awareness of such molecular markers can aid in distinguishing malignant from benign parathyroid lesions and alert clinicians to familial syndromes. Overall, the field is trending toward a more personalized diagnostic approach, integrating traditional pathology with molecular profiling to achieve earlier and more accurate detection of endocrine tumors.

Surgery has historically been—and remains—the cornerstone of curative therapy for most endocrine tumors. Endocrine surgery is generally highly effective given the typically localized nature of these tumors and their often-indolent spread. For instance, in primary hyperparathyroidism (PHPT) caused by benign parathyroid adenoma, surgical removal of the offending gland is the definitive treatment. The contribution to this Special Issue by Migoń et al. reported that parathyroidectomy achieved biochemical cure (normalization of calcium and parathyroid hormone levels) in 95.2% of PHPT patients at six months, with a low complication rate. This underscores that surgery, often minimally invasive, offers excellent outcomes for PHPT and should remain the gold standard for eligible patients. Likewise, in differentiated thyroid cancer, thyroidectomy (often with lymph node dissection) is the primary treatment, frequently followed by radioiodine ablation for remnant or metastatic disease [6].

For certain endocrine tumors, however, surgery can be challenging or insufficient. Pituitary adenomas (e.g., corticotroph adenomas causing Cushing’s disease or somatotroph adenomas causing acromegaly) reside in a sensitive location at the skull base. Transsphenoidal surgery is the first-line treatment for these malignancies, and remission rates for pituitary Cushing’s disease can range from 65 to 90%. Yet even with apparent complete resection, hypercortisolism recurs in up to 30% of patients in the long term, roughly 2% per year. This reality has driven the development of adjunct and alternative therapies. In Cushing’s disease, if surgery fails or is contraindicated, medical therapy targeting the cortisol axis becomes critical. Osilodrostat, a potent inhibitor of adrenal cortisol synthesis, was approved in 2020 as a new medical option for Cushing’s disease. As reported by Popa Ilie et al. in this Special Issue, such adrenal-directed medications can effectively control hypercortisolism [7]. Their study analyzed real-world pharmacovigilance data and highlighted the safety profile of osilodrostat, an important consideration as we increasingly rely on medical management in refractory cases. Other medications (e.g., pasireotide targeting the pituitary tumor; mifepristone as a glucocorticoid receptor blocker) exemplify the growing arsenal of pharmacotherapies addressing endocrine tumors via their hormonal effects. Additionally, radiotherapy (such as stereotactic radiosurgery) can be employed to achieve long-term hormone control when surgery and medications fall short.

Another area of significant progress has been systemic therapy for advanced endocrine malignancies. Traditional cytotoxic chemotherapy has limited efficacy in most well-differentiated endocrine cancers, but targeted therapies and radionuclide therapies are changing the landscape. In aggressive thyroid cancers (poorly differentiated or anaplastic thyroid carcinoma), which have historically had dismal outcomes, molecular targeted therapy is offering new hope. The identification of actionable mutations (like BRAF V600E in roughly half of anaplastic thyroid cancers) has led to targeted drug approvals—for example, combined BRAF/MEK inhibition has produced unprecedented responses in anaplastic thyroid cancer, converting some formerly fatal cases into manageable disease. In medullary thyroid carcinoma, RET proto-oncogene mutations are common, and recently developed RET inhibitors have dramatically improved response rates in advanced cases. These developments were foreshadowed by the molecular insights (e.g., RET mutations) mentioned above and represent how precision oncology is entering endocrinology.

Perhaps the most noteworthy breakthroughs have occurred in the management of metastatic neuroendocrine tumors, which often pose a therapeutic dilemma due to their variable behavior and somatostatin receptor expression. The past few years have seen the advent of peptide receptor radionuclide therapy (PRRT), such as Lutetium-177 DOTATATE, which delivers targeted radiation to NET cells expressing somatostatin receptors and results in prolonged progression-free survival in midgut NETs. In addition, new targeted kinase inhibitors have shown efficacy in NETs. A very recent phase 3 trial (CABINET study) demonstrated that cabozantinib, a multi-tyrosine kinase inhibitor, significantly improves progression-free survival in patients with progressive advanced NETs [8]. In that study, cabozantinib more than doubled median progression-free survival compared to placebo in both pancreatic and extra-pancreatic NET cohorts. This represents a major advance for patients who have exhausted standard options like somatostatin analogs and Everolimus. These “hot” developments in systemic therapy, including trials reported in top journals, are reshaping the treatment algorithms for endocrine tumors. We are now entering an era where multidisciplinary care for endocrine tumors might include not just surgeons and endocrinologists, but also medical oncologists employing targeted drugs and radionuclide therapy specialists delivering radioligand treatments.

Despite these innovations, some endocrine malignancies remain therapeutic challenges. Parathyroid carcinoma is one such example: an ultra-rare cancer where surgery is the only potentially curative modality. There are no established international guidelines or randomized trials for parathyroid carcinoma due to its rarity. As Simescu et al. discuss in their 15-year case series in this Special Issue, management must generally be individualized, and aggressive surgery (en bloc resection) is recommended when feasible, sometimes supplemented by adjuvant radiation or chemotherapy on a case-by-case basis. They underscore that normalizing parathyroid hormone levels via surgery is critical for symptom control and that recurrent disease remains a risk even after apparently successful operations [9]. The lack of standardized protocols for such rare tumors highlights the need for collaborative research and reporting of all clinical experiences to inform future care. In this spirit, the collective evidence presented in this Special Issue—spanning common to rare endocrine tumors—contributes valuable data, helping to guide practitioners around the world.

A defining aspect of endocrine tumors is the need for diligent long-term management. Patients often require lifelong follow-up due to risks of recurrence, metastasis, or delayed effects of hormone excess/deficiency after treatment. As highlighted by several contributions, even “cured” patients benefit from surveillance. For instance, thyroid cancer survivors need periodic ultrasound and thyroglobulin monitoring to catch any recurrence early; in those with more aggressive subtypes, surveillance imaging like PET/CT may be indicated [5]. In Cushing’s disease or acromegaly, patients must be monitored for recurrence of hormonal hypersecretion for years after pituitary surgery—the recurrence rates of ~30% in such conditions mean endocrinologists must remain vigilant. Similarly, long-term biochemical follow-up is paramount after surgery for parathyroid carcinoma, as up to one-third of patients can experience relapse, even after initial remission. The importance of follow-up is a recurring lesson: early detection of a recurrence (be it biochemical or structural) can allow timely re-intervention or therapy adjustment, markedly improving patient outcomes.

Assessing outcomes in endocrine tumor care requires looking beyond traditional survival metrics; quality of life and control of hormone-related symptoms are equally important. Many endocrine tumor patients suffer from comorbidities caused by hormone excess: for example, acromegaly can lead to cardiomyopathy, diabetes, and arthropathy; Cushing’s causes metabolic and cardiovascular complications. Even after tumor removal, these issues may persist and need management. A 2024 cardiac MRI study in *Pituitary* by De Alcubierre et al. demonstrated persistent subclinical cardiac abnormalities in acromegaly patients despite biochemical disease control, directly attributable to prolonged GH/IGF-1 excess [10]. Their findings reinforce that controlling the disease (through surgery or medical therapy) is critical not only to remove the tumor but also to mitigate ongoing end-organ damage. They also underline a broader point: multidisciplinary care involving cardiologists, nephrologists, and other specialists is often required to manage the systemic sequelae of endocrine tumors. Thus, “successful treatment” of an endocrine tumor is not just about eradicating the tumor, but also about managing the hormonal milieu and rehabilitating the patient’s overall health.

Encouragingly, for several endocrine neoplasms, long-term outcomes are improving. Differentiated thyroid carcinoma, for instance, generally has an excellent prognosis, with over 95–98% 10-year survival for early-stage disease. Even some NETs, which were once synonymous with relentless progression, can have surprisingly favorable outcomes. Serafin et al. reported on a decade of experience with gastrointestinal neuroendocrine neoplasms (GI-NENs) in this Special Issue and found that, despite most patients presenting with advanced disease (over 45% had distant metastases), the five-year overall survival was about 95% in their cohort. This high survival rate speaks to the effectiveness of modern multimodal therapy and perhaps the indolent nature of many well-differentiated NETs. Their study also noted that midgut (small intestinal) NETs were the predominant subtype, and that surgical resection, often including metastasectomy when possible, likely contributed to the positive outcomes. It is worth noting, however, that survival statistics for NETs vary widely by grade and stage; well-differentiated, slow-growing tumors can often be managed chronically, whereas high-grade neuroendocrine carcinomas still carry a poor prognosis. The heterogeneity of NETs once again underscores the need for personalized approaches and ongoing research.

In the case of aggressive endocrine malignancies like anaplastic thyroid carcinoma or high-grade NETs, the outlook remains guarded, but even here there are glimmers of progress. Subbiah et al. reported that targeted therapy with combined BRAF/MEK inhibitors produced a 69% response rate in BRAFV600E-mutant anaplastic thyroid carcinoma [11]. The authors emphasize the importance of recognizing these variants and tailoring treatment, often resulting in an aggressive multimodal approach combining surgery, radiation, and systemic therapy. Recent years have seen the addition of targeted therapies (as mentioned earlier) and even immunotherapies being tested in trials for these refractory cases. While a cure may remain elusive for many patients with the most aggressive endocrine tumors, incremental improvements in therapy are hopefully extending survival and certainly improving symptom control.

The collection of studies in this Special Issue highlights the dynamic and multidisciplinary nature of modern endocrine tumor management. We have seen how cutting-edge imaging techniques, molecular diagnostics, and targeted treatments are being applied to conditions that were once managed with a one-size-fits-all approach. Importantly, this Special Issue’s contributions span the spectrum from common diseases like thyroid nodules to rare malignancies like parathyroid carcinoma, reflecting a broad effort to advance knowledge across the field. What connects these works is their shared goal of improving patient outcomes through better understanding of disease behavior and thoughtful integration of new technologies into clinical practice.

Several overarching themes emerge from this body of research. First, early and precise diagnosis is the foundation. Whether via improved risk stratification of thyroid nodules to avoid unnecessary surgery or via genetic testing to unmask hereditary syndromes, identifying the nature of the tumor promptly enables optimal treatment planning. Second, surgical management remains curative in most localized endocrine tumors, and its techniques continue to be refined (for example, minimally invasive endocrine surgery, intraoperative PTH monitoring, endoscopic approaches to the skull base, etc.). Third, innovation in therapy—from pharmacological breakthroughs like osilodrostat for Cushing’s and cabozantinib for NETs to advanced radionuclide therapies—is expanding options for patients with advanced or recurrent endocrine tumors, offering hope in situations that were previously dire. Fourth, collaboration and data-sharing are crucial, especially for rare tumors. As noted in the parathyroid carcinoma study, every documented case series adds to our collective knowledge in the absence of large trials. This Special Issue itself serves as a collaborative platform, bringing together insights from different centers and specialists around the world.

Looking forward, the management of endocrine tumors is poised to become even more personalized. Ongoing research into tumor genomics and the tumor microenvironment may yield novel targets for therapy (for instance, immunotherapy approaches for endocrine cancers are under investigation, and gene-specific therapies continue to emerge). There is also a growing emphasis on quality of life and survivorship, ensuring that patients not only live longer but live better, free of the debilitating effects of hormone excess or deficiency. Future studies will likely focus on integrating patient-reported outcomes and endocrine-specific quality metrics into assessments of treatment success.

As Guest Editors of this Special Issue, we are encouraged by the advancements captured in these articles and their potential impact on clinical practice. The dialogue between research and clinical care is clear: clinical challenges are driving innovative research, and research findings are rapidly being translated into new standards of care. We hope that readers will find these studies thought-provoking and useful in guiding their own practice or research. Ultimately, the collective efforts of the endocrine tumor community—endocrinologists, surgeons, oncologists, pathologists, nuclear medicine physicians, and scientists—is bringing us closer to the shared objective of better outcomes for patients affected by endocrine tumors. By continuing to foster collaboration and disseminate new knowledge through forums like this Special Issue, we aim to advance the field further. In closing, we thank all the contributors and reviewers involved in this Special Issue for helping to illuminate the path forward in diagnosis, treatment, and management of endocrine tumors, and we look optimistically toward the future developments that will build upon this foundation.
